# Supplementary material for: Path analysis of factors associated with nurses’ pain management practices in older adults with cognitive impairment: A cross-sectional study
Source: PLoS One. 2026 Jun 1;21(6):e0349931. doi: 10.1371/journal.pone.0349931 (PMC13225366; doi:10.1371/journal.pone.0349931)
Supplement: Attachment — Submitted filename: PLOS revision.docx [file pone.0349931.s001.docx]

**Overall significance**

- The manuscript examines a significant and underexplored domain: the pain management practices of nurses concerning older adults with cognitive impairment, employing a theoretically informed path analysis. The integration of Social Cognitive Theory and the analysis of self-efficacy as a mediator significantly contributes to nursing and gerontological pain literature, especially in the Thai context.
- The manuscript requires professional English language editing.
- The references are up-to-date and appropriate for publication.
- Clarity and Structure: The manuscript is well organized and clearly articulated. Certain sections would benefit from enhanced conciseness.

**Abstract**

It is advisable to briefly indicate the cross-sectional and secondary analysis characteristics of the study in the Methods section to improve transparency.

**Background**

- The background offers a compelling rationale, underpinned by pertinent and current literature.
- The transition from prevalence to clinical consequences to nursing challenges is coherent.

**Suggestions:**

- Some paragraphs (pages 11–12) are dense and could be streamlined by reducing overlapping citations.
- Clearly identify the existing knowledge gap by noting that previous path analyses or mediation models have not investigated these variables in older adults with cognitive impairments.
- Explicitly reference and interpret Figure 1 in relation to the hypothesized paths.

**Methods**

- **Study Design:** Add a brief justification for why secondary analysis was suitable for addressing the current research aim.
- **Tools:** Clarify how adding new items to TENAP Part B may affect comparability with prior studies.
- **Data Analysis:** The statistical approach is appropriate and well justified.
- Briefly state how assumptions for SEM (normality, multicollinearity) were assessed.

**Results:**

- The results are presented clearly and sequenced logically.

**Discussion:**

- The discussion is clearly articulated, and the interpretation of self-efficacy as a mediator is notably robust.
- Reduce redundancy in the discussion of knowledge, collaboration, and experience throughout various paragraphs.
- To improve international relevance, explicitly compare findings with non-Asian or Western contexts.

**Limitations:**

- Add a brief note on common method bias due to self-report measures.

**Conclusion**:

- Refrain from repeating detailed findings; rather, highlight the significance of noting essential points.
