# Supplementary material for: Path analysis of factors associated with nurses’ pain management practices in older adults with cognitive impairment: A cross-sectional study
Source: PLoS One. 2026 Jun 1;21(6):e0349931. doi: 10.1371/journal.pone.0349931 (PMC13225366; doi:10.1371/journal.pone.0349931)
Supplement: Attachment — Submitted filename: Respond to reviewers.docx [file pone.0349931.s002.docx]

**PLOS One: PONE-D-25-58744**

**Path analysis of Factors Associated with Nurses’ Pain Management Practice in Older Adults with Cognitive Impairment: a cross-sectional study**

| **Editor Comments** | **Author’s response** |
| --- | --- |
| **Overall significance**  • The manuscript examines a significant and underexplored domain: the pain management practices of nurses concerning older adults with cognitive impairment, employing a theoretically informed path analysis. The integration of Social Cognitive Theory and the analysis of self-efficacy as a mediator significantly contributes to nursing and gerontological pain literature, especially in the Thai context.  • The manuscript requires professional English language editing.  • The references are up-to-date and appropriate for publication.  • Clarity and Structure: The manuscript is well organized and clearly articulated. Certain sections would benefit from enhanced conciseness. | Thank you so much for the positive and overall evaluation of our manuscript. Regarding the English language quality, the manuscript has been edited by a native speaker to improve clarity and readability throughout according to your comment. |
| **Abstract**  It is advisable to briefly indicate the cross-sectional and secondary analysis characteristics of the study in the Methods section to improve transparency. | Thank you so much for your feedback. We have added a justification for the use of secondary data analysis as follows:  Study Design: The secondary analysis was aligned with the study objectives and the original data collection purpose and was supported by validated measurement tools and an adequate sample size.  A justification has been added to both the abstract and the main text. |
| **Background**  **•** The background offers a compelling rationale, underpinned by pertinent and current literature.  • The transition from prevalence to clinical consequences to nursing challenges is coherent. | Thank you so much for your positive feedback. |
| **Suggestions:**  **•** Some paragraphs (pages 11–12) are dense and could be streamlined by reducing overlapping citations.  • Clearly identify the existing knowledge gap by noting that previous path analyses or mediation models have not investigated these variables in older adults with cognitive impairments.  • Explicitly reference and interpret Figure 1 in relation to the hypothesized paths. | Thank you for your insightful suggestion!  We have reduced overlapping citations and prioritized recent references throughout the background section. However, Bandura’s foundational theory of self-efficacy was retained, as it is essential to the conceptual framework. These revisions improve clarity and coherence while preserving theoretical integrity. |
| **Methods**  • **Study Design:** Add a brief justification for why secondary analysis was suitable for addressing the current research aim.  • **Tools:** Clarify how adding new items to TENAP Part B may affect comparability with prior studies.  • **Data Analysis:** The statistical approach is appropriate and well justified.  • Briefly state how assumptions for SEM (normality, multicollinearity) were assessed.  **Results:**  • The results are presented clearly and sequenced logically. | Thank you so much for your feedback. We have added a justification for the use of secondary data analysis as follows:  Study Design: The secondary analysis was aligned with the study objectives and the original data collection purpose and was supported by validated measurement tools and an adequate sample size.  A justification has been added to both the abstract and the main text.  **Tools:** We add a clarification on page 6, second paragraph as follows:  As no existing instrument specifically evaluates pain management in older adults with cognitive impairment, we reviewed the literature and obtained approval from the developer to expand the instrument. Thus, eleven additional items were added to each vignette to assess nurses’ pain management practices in this population. Following the development, the Thai questions were submitted to five Thai experts for feedback on clarity, relevance, and logical flow.  **Data analysis:**  Thank you for your suggestion. We have included how SEM assumptions were assessed as follows:  Page 8, paragraph 2 under Data analysis:  Variance inflation factors (VIF), skewness, and kurtosis were assessed to evaluate potential violations of multicollinearity and normality assumptions. In this study, all VIF values were below 10, indicating no evidence of problematic multicollinearity among the independent variables. However, the distributions of years of nursing experience and nurses’ pain management practice scores demonstrated notable skewness, suggesting deviation from normality.  Page 8, paragraph 4: Path analysis was conducted using structural equation modeling (SEM). To estimate the coefficients of direct effects and to test mediation effects, we employed a bootstrapping method with 5,000 resamples; bootstrapping is recommended for evaluating the significance of indirect effects as it does not rely on the assumption of normality of the sampling distribution and provides more accurate confidence intervals, therefore reducing the risk of Type I error (36).    **Results:** Thank you so much for your feedback. |
| **Discussion:**  **•** The discussion is clearly articulated, and the interpretation of self-efficacy as a mediator is notably robust.  • Reduce redundancy in the discussion of knowledge, collaboration, and experience throughout various paragraphs.  • To improve international relevance, explicitly compare findings with non-Asian or Western contexts. | Thank you so much for your feedback. We reviewed the entire manuscript and minimized redundancy throughout. However, as research on pain management among older adults with cognitive impairment remains limited, we were only able to identify a small number of comparable studies conducted in Asian populations. We hope the current comparisons are information and appropriate to be considered |
| **Limitations:**  • Add a brief note on common method bias due to self-report measures. | Thank you for your comment. We acknowledge that the use of self-report questionnaires may introduce common method bias, which cannot be fully eliminated and should be carefully considered when interpreting the findings. To minimize this issue in our study, we assured anonymity and confidentiality of participants as well as used clear and neutral wording throughout the questionnaire. A statement below has now been added to the limitation section.  Moreover, common method bias due to self-report measures may increase the risk of social desirability bias. Therefore, future studies are encouraged to use multi-source data collection (i.e., observational measures such as checklists) to minimize its potential impact. |
| **Conclusion:**  **•** Refrain from repeating detailed findings; rather, highlight the significance of noting essential points. | Thank you so much for your valuable suggestion. We have revised our conclusion as follows:  The findings underscore the importance of nurses’ knowledge and attitudes, collaborative perceptions, years of experience, and pain management self-efficacy in shaping pain management practices. This information highlights the need for targeted interventions and policy development to improve pain management for older adults with cognitive impairment, a vulnerable population in Thailand. |

| **Reviewer 2 Comments** | **Author’s response** |
| --- | --- |
| Objective does not state clearly what is the goal of study?, what research will achieve/outcome after finding relationships. it is not clear. the study needs more strong need assessment as to what happen due to limited knowledge of pain management in terms of cognitive impairment elderly clients?, Discuss factors behind direct and indirect relation among variables and impacts in quantifiable data, more studies from different countries is required in discussion. adding the impact is crucial in discussion. recommendation is required in separate heading, the topic is vital to understand and explore more on pain management, appreciated to develop the research. the study can also explore views from patient their perceptions to relate further the impact. | Thank you so much for your feedback. We have revised the objective and finding outcome to make it clearer as follows:  **Background:** Pain management is a fundamental component of patient care. Inadequate pain management in patients is associated with increased anxiety, depression, and reduced quality of life. Several studies assessed the nurse’ pain management practice in Thailand; however, research focusing on older adults with cognitive impairment remains limited. Therefore, the study aimed to investigate relationships among nurses’ knowledge and attitude, collaborative perception, years of experience, pain management self-efficacy, and their pain management practices.  **Conclusions:** The findings…... Thus, targeted intervention, structured in-service training programs, and supportive policy development should be initiated to enhance pain management competencies and improve the quality of nursing care among older adults with cognitive impairment. |

| **Reviewer 3 Comments** | **Author’s response** |
| --- | --- |
| This study focuses on nurses' pain management practices in this population, which closely aligns with clinical needs and holds practical relevance. However, the following areas still require improvement: | Thank you so much for your feedback. |
| 1.Inconsistent use of terminology: For example, “collaborative perception” and “perception of collaboration” are used interchangeably. It is recommended to unify the terms throughout the manuscript. | Thank you so much for your valuable comment. After carefully reviewing the manuscript, we have revised the terminology throughout to consistently use the term “perception of collaboration” to ensure clarity and alignment with the measurement tool, as this terminology reflects how nurses perceive their collaboration with physicians. |
| 2. The model explained only 37% of the variance. It is suggested that the discussion briefly addresses other potential factors influencing pain management practices, such as organizational culture and resource support. | Thank you so much for your comment. We have added further clarification and supporting information as follows:  Page 17, last paragraph:  Pain management is a fundamental component of effective nursing care, serving as a nursing-sensitive indicator and a continuous nursing-focused process that reflects the quality of pain interventions (45, 46). However, pain management practices are complex and associated with multiple factors. While this study identified several significant individual-level factors, a substantial proportion of variance in pain management practices remained unexplained. Prior studies have shown that pain management is related not only to individual characteristics but also to organizational factors (47, 48). Therefore, systemic conditions within the Thai healthcare context warrant further investigation. Such efforts may inform the development of hospital-level policies and ultimately enhance nurses’ competencies in pain management. |
| 3. The findings support that improving nurses’ self-efficacy can enhance pain management practices. It is recommended to explicitly propose targeted training strategies in the “Conclusion” or “Recommendations” section, such as scenario-based simulation and reflective practice. | Thank you for your suggestion. We have revised our conclusion as follows:  5. Conclusion  The findings underscore the importance of pain management self-efficacy in relation to pain management practices. These results highlight the need for targeted interventions and training strategies such as scenario-based simulation and reflective practice along with supportive institutional policies, may help strengthen pain management for older adults with cognitive impairment. |
| 4. All participants were female. It is suggested that this limitation be further explained in the discussion, and future studies should consider including male nurses or nursing populations with different educational backgrounds. | Thank you for your comment. We have now updated the limitations sections as follows:  **4.1 Limitations**  This study has several limitations that should be acknowledged. First, a cross-sectional design was used, which could not demonstrate causality. Future longitudinal studies are needed to better explain the relationships among the variables. Second, the study was conducted in Thailand, where 98.7% of the nursing workforce is female (49). Thus, the findings may not be generalized to other geographic regions and cultural contexts. Replicating of this study in primary and secondary care hospitals mor among male nurses is recommended to confirm the findings. Finally, the use of self-report measures may introduce common method bias and increase the risk of social desirability bias. Future studies are encouraged to incorporate multi-source data collection (i.e., observational measures such as checklists) to minimize this potential impact. |

| **Reviewer 4 Comments** | **Author’s response** |
| --- | --- |
| This manuscript addresses an important and underexplored topic: factors associated with nurses’ pain management practices for older adults with cognitive impairment. The topic is clinically relevant, theoretically grounded, and aligned with PLOS ONE’s scope. The use of Social Cognitive Theory and path analysis provides a coherent conceptual framework, and the sample size appears adequate for the proposed analyses. Overall, the study has merit; however, several issues should be addressed to improve clarity, rigor, and transparency before the manuscript can be considered for publication. | Thank you so much for your kindly feedback. |
| **Major comments**  The Introduction would benefit from structural consolidation. Currently, background information, literature review, and conceptual framing are distributed across multiple sections with some repetition. These sections could be merged into a single, more concise Introduction that progresses from (1) the clinical importance of pain management in older adults with cognitive impairment, to (2) existing evidence on nurses’ knowledge, attitudes, collaboration, and experience, and (3) the theoretical grounding in Social Cognitive Theory leading to the study aim. This restructuring would improve flow, reduce redundancy, and enhance readability without loss of content. | Thank you for your suggestion. We have revised and reorganized the Introduction to improve flow and eliminate repetition in accordance with your feedback. This restructuring now enhances coherence and readability without losing content. |
| **Conceptual justification of the model**  The manuscript states that Social Cognitive Theory (SCT) guided the model; however, only a subset of SCT constructs (knowledge/attitudes, collaboration, self-efficacy) are operationalized. Other core SCT components such as outcome expectations, observational learning, and reinforcement are not included. The authors should strengthen the theoretical justification explaining why the selected constructs were prioritized and how collaboration and years of experience conceptually map onto SCT domains. This clarification would strengthen the conceptual coherence of the model. | Thank you so much for your feedback. Our study focused on components that are directly related to nurses’ pain management practices in clinical settings; therefore, only theoretically relevant components were included. Below our revisions:   1.1 **Conceptual Framework**   This study was guided by Social Cognitive Theory (SCT) and relevant literature review. Social Cognitive Theory, developed by Albert Bandura, describes how personal, behavioral, and environmental factors interact to influence individual behaviors (19). Although SCT includes multiple key components (i.e., self-efficacy, outcome expectations, observational learning, and reinforcement), this study focused on those most directly related to nurses’ pain management practices in clinical settings (19). Thus, only theoretically relevant components were operationalized.  Self-efficacy, one of the key elements of SCT, refers to an individual’s belief in their ability to perform specific tasks, influencing behavior change or practice (19). According to Bandura, self-efficacy is influenced by personal factors such as knowledge, attitudes, and expectations, along with behaviors, and environmental factors like peers and family (19, 20). Knowledge and attitudes were conceptualized as cognitive determinants that contribute to the development of self-efficacy (19). Individuals with high self-efficacy are more likely to engage in and perform desired behaviors (19, 21).  Years of experience were viewed as a source of mastery experience that may strengthen self-efficacy, while nurses’ perceptions of collaboration with physicians were considered as environmental influences (15, 16). Both factors are associated with self-efficacy and pain management (15, 16). However, evidence regarding these relationship in older adults with cognitive impairment remains limited. Therefore, examining the relationships among these factors is warranted. See **Figure 1** for the study’s theoretical framework details. |
| **Model modification and path removal**  The manuscript notes that the initial model was just-identified and that the path from years of experience to pain management practice was removed to improve model fit. This modification requires clearer justification. Please clarify whether this decision was theory-driven or guided by model diagnostics (e.g., modification indices). Post-hoc model changes should be explicitly justified to avoid concerns about data-driven model fitting. | Thank you for your comment. As the direct path from years of experience to pain management practice was found to be statistically non-significant. The model was respecified by removing this path to achieve parsimony and to align with SCT, which conceptualizes years of experience as contributing to behavior through self-efficacy rather than as a direct predictor. Below is our revision version.  **3.2 Testing the model of nurses’ pain management practice in older adults with cognitive impairment**  Because the hypothesized model was a just-identified model with zero degrees of freedom, the fit indices could not be evaluated. The initial model was refined by removing this path from years of experience to pain management practice to achieve parsimony while maintaining alignment with theory and previous empirical evidence. Within SCT, years of experience is conceptualized as contributing to behaviors through self-efficacy rather than as a direct predictor (16, 17, 33). As shown in **Figure 2**, the final model showed adequate fit to the data: χ^2^ = 1.642 (*df* = 1, *p* = 0.200), TLI = 0.956, CFI = 0.994, RMSEA = 0.061, and SRMR = 0.019. The final model accounted for 37% of the variance in pain management practice. |
| **Interpretation of mediation using cross-sectional data**  Although bootstrapped mediation was conducted appropriately, the cross-sectional design limits causal inference. Throughout the Results and Discussion, causal wording such as “influences,” “improves,” or “leads to” should be softened and replaced with language such as “is associated with” or “is consistent with an indirect relationship.” This clarification is important to avoid overstating causal conclusions. | Thank you so much for raising this critical point. We have removed causal words from the Result and Discussion sections in accordance with your suggestion. |
| **Measurement considerations and self-report bias**  All key variables, including pain management practice, were measured using self-report instruments. This raises the possibility of social desirability and common-method bias. While this limitation is acknowledged, it should be more explicitly emphasized. The authors are encouraged to discuss how future studies could incorporate observational measures, audits, or objective indicators of practice. | Thank you for your suggestion. We have added the limitation related to self-report measures as describe below:  Moreover, common method bias due to self-report measures may increase the risk of social desirability bias. Therefore, future studies are encouraged to use multi-source data collection (i.e., observational measures such as checklists) to minimize its potential impact. |
| **Modification of the TENAP instrument**  The manuscript describes substantial adaptation of the TENAP instrument, including adding new items and expanding its scope from assessment to management. While psychometric testing is reported, the authors should more clearly acknowledge that this represents an expanded or modified version of the original instrument. Clarification is needed regarding construct equivalence and comparability with prior TENAP-based studies. | Thank you so much for your valuable comment. We acknowledge that this study used a modified and expanded version of the TENAP instrument to include pain management items, as no existing tool specifically assesses both pain assessment and management in older adults with cognitive impairment. Although a prior study by Alomari et al. (2021) included pain management items, this instrument was designed for general older adults rather than those with cognitive impairment. So, adaptation was necessary to ensure content relevance to older adults with cognitive impairment.  We have clarified this adaptation in the revised manuscript and noted that direct comparability with prior TENAP may be limited.  As no existing instrument specifically evaluates pain management in older adults with cognitive impairment, we reviewed the literature and obtained approval from the developer to expand the instrument. Thus, eleven additional items were added to each vignette to assess nurses’ pain management practices in this population. Following the development, the Thai questions were submitted to five Thai experts for feedback on clarity, relevance, and logical flow.  However, direct comparability with previous TENAP-based studies may be limited due to these modifications. |
| **Statistical reporting clarity**  Please clarify whether coefficients reported in tables and figures are standardized or unstandardized.  Ensure consistency in reporting correlation coefficients (Pearson vs. Spearman) and notation.  Some confidence intervals appear wide relative to the reported coefficients and should be double-checked.  Minor formatting inconsistencies (spacing, p-value notation) should be corrected. | Thank you for your questions.  All coefficients reported in the table and figures are standardized coefficients.  Also, Spearman’s rank-order correlation was used throughout the analysis.  In addition, we corrected minor formatting inconsistencies (i.e., spacing and p-value notation) to ensure consistency throughout.  Regarding the confidence intervals, we have checked, recalculated them, and revised the manuscript where necessary. |
| **Generalizability**  The study sample consists entirely of female nurses from two hospitals in Thailand. While this reflects the local workforce context, the authors should more clearly acknowledge limits to generalizability beyond similar cultural and healthcare settings.or nursing populations with different educational backgrounds. | Thank you for your suggestion. We have added the following statement to clarify the generalizability of the findings:  This study has several limitations that should be acknowledged. First, a cross-sectional design was used, which could not demonstrate causality. Future longitudinal studies are needed to better explain the relationships among the variables. Second, the study was conducted in Thailand, where 98.7% of the nursing workforce is female (49). Thus, the findings may not be generalized to other geographic regions and cultural contexts. Replicating of this study in primary and secondary care hospitals mor among male nurses is recommended to confirm the findings. Finally, the use of self-report measures may introduce common method bias and increase the risk of social desirability bias. Future studies are encouraged to incorporate multi-source data collection (i.e., observational measures such as checklists) to minimize this potential impact. |
| **Minor comments**  **-**Minor grammatical and stylistic issues are present throughout the manuscript and should be corrected during revision.  -Keywords should be standardized (e.g., “nurses’ pain management” rather than “nurse’ pain management”).  -Figures would benefit from clearer labeling and explicit indication of standardized coefficients.  -Some repetition in the Discussion could be reduced to improve clarity and conciseness. | Thank you for your helpful feedback. We have carefully reviewed the manuscript and fixed grammatical and stylistic issues in the revised version. We have also revised the keywords and figure accordingly. Also, the discussion section was reviewed, and repetitive content has been removed. |
| **Summary**  This study addresses a relevant clinical and educational issue and applies an appropriate analytical approach. With clearer theoretical justification, more cautious interpretation of mediation effects, improved reporting transparency, and minor editorial revisions, the manuscript would be suitable for publication. | We truly appreciate your encouraging feedback. The team has carefully revised the entire manuscript to ensure that it meets publication standards and contributes meaningfully to research and nursing practice. |
