# Supplementary material for: Path analysis of factors associated with nurses’ pain management practices in older adults with cognitive impairment: A cross-sectional study
Source: PLoS One. 2026 Jun 1;21(6):e0349931. doi: 10.1371/journal.pone.0349931 (PMC13225366; doi:10.1371/journal.pone.0349931)
Supplement: Attachment — Submitted filename: Responses to reviewer.docx [file pone.0349931.s003.docx]

Path analysis of Factors Associated with Nurses’ Pain Management Practices in Older Adults with Cognitive Impairment: a cross-sectional study

| **Review Comment to the Author** | **Author response to the reviewer** |
| --- | --- |
| **Abstract:** Keywords are not fully aligned with MeSH terminology. | Thank you for your valuable feedback. We have revised the keywords to ensure alignment with MeSH terminology. The updated keywords are: Cognitive dysfunction; collaboration; nurses; knowledge; pain management; self-efficacy |
| **Literature Review and Conceptual Framework:** Figure 1 is not sufficiently explained or integrated into the text. | Thank you for the comment. We have revised the manuscript to better explain and integrate Figure 1 into the text, including clarification of the conceptual framework and hypothesized relationships. These revisions have been incorporated on **page 4.** |
| **Methods**  - Participant flow, inclusion/exclusion criteria, and response rate are not clearly reported.  - No a priori sample size calculation or statistical power analysis is clearly justified. | Thank you so much for your feedback. We have revised the Methods section to clarify the participant flow, inclusion and exclusion criteria, and response rate. In addition, a prior sample size calculation and statistical power analysis have been included. These revisions are presented on **page 5.** |
| **Measurement Validity and Reliability**  1. Scoring procedures and interpretation of scale direction are inconsistently described.  2. The modification of the TENAP instrument is insufficiently justified in terms of construct validity.  3. The comparability with previous studies using the original instrument is not adequately addressed.  4. The manuscript does not report the presence, proportion, or handling of missing data. | **1.** We have revised the Methods section to clarify the scoring procedures and ensure consistency in the interpretation of scale direction for the TENAP (pages 6-7) and CSACD (**page 8**). Specifically, we clarified item scoring, Likert scale ranges, and the interpretation to total scores.  **2.** We have revised the manuscript to strengthen the justification for the modification of the TENAP instrument by providing additional evidence of construct validity. We examined convergent and divergent validity, showing a moderate positive correlation with nurses’ pain management self-efficacy and no significant correlation with work complexity. These revisions have been added on **page 8.**  **3.** We have revised the manuscript to clearly describe the differences between the original and modified TENAP and to clarify the implication for comparability. We added a paragraph explaining that the original TENAP assessed nurses’ knowledge and attitudes toward pain management and pain assessment, whereas the modified version expands the instrument to include pain management practices, resulting in both conceptual and structural differences (additional items and an expanded scoring range). We also clarified that these differences may limit direct comparability with previous-based studies. These revisions have been added on **page 7.**  **4.** We reported how we handled missing data on **pages 5 and 6.** Specifically, questionnaires were checked for completeness upon return, and participants were given the option to complete any accidentally missed items or submit the questionnaire without modification. Initial screening identified minimal missing data, and all participants chose to complete the missing items, resulting in a final dataset with no missing data. |
| **Results**  1. Descriptive statistics lack consistent formatting across tables.  2. Tables do not consistently include footnotes explaining scale ranges and score interpretation.  3. Percentages are inconsistently reported (decimal places vary).  4. Table titles do not consistently report sample size (N).  5. Variable labels are inconsistently formatted.  6. Use of commas instead of decimal points violates international reporting standards.  7. Structural model results are presented clearly; however, confidence intervals and effect sizes are not fully integrated into the narrative.  8. The non-significant paths are not adequately contextualized.  9. The explained variance (37%) is not critically interpreted.  **Discussion**  1. Direct and indirect relationships are not sufficiently explained mechanistically.  2. Limited comparison with international (non-Asian) studies reduces global relevance.  3. Some statements approach causal interpretation despite a cross-sectional design.  4. Residual redundancy is present across discussion sections. | Thank you for these helpful suggestions. We have revised all tables to address the issues raised **(points 1–5).** Specifically, we standardized the formatting of descriptive statistics, added footnotes explaining scale ranges and score interpretation, ensured consistent reporting of percentages (uniform decimal places), included sample sizes (N) in all table titles, and revised variable labels for consistency throughout the manuscript.  **6**. We have revised all numerical values throughout the manuscript to ensure the use of decimal points according to international reporting standards.  **7.** We have revised the results section to fully integrate confidence intervals and effect sizes into the narrative description of the structural model findings. In addition, confidence interval values have been corrected and are now constituently reported alongside effect sizes to provide a complete interpretation of the results. The revision has been made on pages 12-13.  **8.** We have revised the Results section to better contextualize the non-significant paths by providing an interpretation of their implications on page 12.  **9.** We have revised the manuscript to better interpret the explained variance, as follows: The final model accounted for 37% of the variance in nurses’ pain management practices (R^2^ = 0.37), indicating that the model explains a moderate amount of variance. The remaining 63% suggests that additional factors not included in the model may influence pain management practices.  Thank you so much for your feedback. We have revised the Discussion section to improve clarify and interpretation across the section. We added more comparisons with international studies to enhance global relevance. We also revised wording throughout to avoid causal interpretations. Also, redundant statements were reduced to improve clarity and conciseness. |
| **Implications and Recommendations**  1. A separate “Recommendations” section is not provided despite the reviewer’s request.  2. Practical implications remain general and lack actionable strategies.  3. Clinical, educational, and policy-level implications are not clearly differentiated. | Thank you for your valuable suggestion. We have added a separate “Implications and Recommendations” section and revised the content to provide more specific and actionable strategies across clinical, educational, and policy levels in response to your feedback. |
| **References**  1. The reference list does not consistently follow the journal’s required citation style.  2. The manuscript relies heavily on region-specific sources, limiting global applicability. | Thank you for these helpful comments. We have reviewed and revised all references to ensure consistency with the journal’s citation style. Also, we have included additional international studies from diverse regions to enhance the global relevance and strengthen the comparative context of the manuscript. |

**Note:** We have also revised the conclusion sections in both the abstract and the main text to ensure alignment.
